# The Selective Autophagy Receptor Optineurin in Crohn’s Disease

**DOI:** 10.3389/fimmu.2018.00766

**Published:** 2018-04-10

**Authors:** Markus Tschurtschenthaler, Timon Erik Adolph

**Affiliations:** ^1^Center for Translational Cancer Research (TranslaTUM), Technical University of Munich, Munich, Germany; ^2^Department of Internal Medicine II, Klinikum Rechts der Isar, Technical University of Munich, Munich, Germany; ^3^Department of Medicine I (Gastroenterology, Endocrinology and Metabolism), Medical University Innsbruck, Innsbruck, Austria

**Keywords:** optineurin, inflammatory bowel disease, Crohn’s disease, endoplasmic reticulum stress, selective autophagy

## Abstract

Autophagy is a pathway that allows cells to target organelles, protein complexes, or invading microorganisms for lysosomal degradation. The specificity of autophagic processes is becoming increasingly recognized and is conferred by selective autophagy receptors such as Optineurin (OPTN). As an autophagy receptor, OPTN controls the clearance of *Salmonella* infection and mediates mitochondrial turnover. Recent studies demonstrated that OPTN is critically required for pathogen clearance and an appropriate cytokine response in macrophages. Moreover, OPTN emerges as a critical regulator of inflammation emanating from epithelial cells in the intestine. OPTN directly interacts with and promotes the removal of inositol-requiring enzyme 1α, a central inflammatory signaling hub of the stressed endoplasmic reticulum (ER). Perturbations of ER and autophagy functions have been linked to inflammatory bowel disease (IBD) and specifically Crohn’s disease. Collectively, these studies may explain how perturbations at the ER can be resolved by selective autophagy to restrain inflammatory processes in the intestine and turn the spotlight on OPTN as a key autophagy receptor. This review covers a timely perspective on the regulation and function of OPTN in health and IBD.

## Introduction

Autophagy is an evolutionary conserved self-cannibalistic pathway that leads to the degradation of bulk cytoplasm (macroautophagy) in order to generate energy and to maintain cell homeostasis (1). However, researchers are increasingly appreciating that receptors specifically guide autophagic degradation as exemplified by the removal of damaged organelles [e.g., mitophagy of mitochondria, ER-phagy of endoplasmic reticulum (ER), pexophagy of peroxisomes], bacteria (xenophagy), lipid droplets (lipophagy), protein aggregates (aggrephagy), and other cytosolic constituents (2, 3). This rather selective autophagic process may be controlled by Optineurin (OPTN) besides other autophagy receptors including p62/SQSTM1, NBR1, CALCOCO2/NDP52, and TAX1BP1 (3, 4). These receptors recognize ubiquitinated cargo via their ubiquitin-binding domains (UBA, UBZ or UBAN) and tether it to the autophagosomal membranes by their LC3-interacting regions (LIRs) (5). However, OPTN does not only guide selective autophagy but also controls tumor necrosis factor (TNF), nuclear factor κB (NF-κB), and type I interferon (IFN) signaling (6–8). OPTN has been implicated in a variety of human diseases including glaucoma (9), amyotrophic lateral sclerosis (10, 11), Paget’s disease (12, 13), and recently, inflammatory bowel disease (IBD) (14, 15). IBD comprise a spectrum of complex diseases that affect the gastrointestinal tract and organs beyond the intestine (e.g., eye, skin, joints). IBD is clinically distinguished into two major phenotypes: ulcerative colitis (UC) and Crohn’s disease (CD). Although these two diseases share some genetic risk they are considered separate disease entities due to their localization, clinical presentation and response to therapy (16). The pathophysiology of these diseases involves environmental factors and their impact on the intestinal microbiota which may orchestrate a chronic remittent form of inflammation in genetically susceptible hosts (17–19). Genetic variation in the autophagy gene *ATG16L1* has been linked to CD (20, 21) which leads to an impaired autophagic response due to caspase 3-mediated cleavage of the mutant ATG16L1 variant (22). Impaired autophagy function especially in intestinal epithelial cells results in the susceptibility to small and large intestinal inflammation (23–27). As such, it is conceivable that the selective autophagy receptor OPTN may regulate inflammatory processes in the intestine. Evidence for the regulation and function of OPTN in intestinal inflammation and specifically IBD is covered in this review.

## The Selective Autophagy Receptor OPTN in Health

The gene encoding OPTN is evolutionary conserved and expressed in most tissues of the human body (28). OPTN was initially discovered in 1998 in a yeast two-hybrid screen as a binding partner of the adenovirus protein E3-14.7K (early region 3 of group C human adenoviruses 14.7 kDa), and was thereafter named as FIP-2 (for 14.7 kDa interacting protein) (28). Later, this gene was identified to have a strong homology with NEMO (NF-κB essential modulator) and was subsequently denoted as NEMO-related protein (29). But it also became known as transcription factor IIIA-interacting protein, Huntingtin-interacting protein 7, and Huntingtin yeast partner L (30). Eventually, the multifunctional protein was renamed to OPTN (“optic neuropathy inducing”), as it was found to play a major neuroprotective role and mutations in this gene were shown to be causative for the development of primary open-angle glaucoma, a leading cause of blindness (9).

The human *OPTN* gene is located at chromosome 10 and consists of three non-coding exons in the 5′UTR and 13 exons that encode a 577 amino acid protein with a size of 66 kDa. The mouse *Optn* gene is located at chromosome 2 and also contains 13 exons, which encodes a full-length protein of similar size that shows 78% sequence similarity to the human protein (6, 30). The OPTN protein consists of several functional domains including a basic leucine zipper motif (bZIP), a microtubule-associated protein 1 light-chain LIR, a ubiquitin-binding domain (UBAN), multiple coiled-coil motifs as well as a ubiquitin-binding zinc-finger domain at the C-terminus (6). Notably, NEMO, a central regulator of NF-κB activation shares 53% similarity with OPTN and only lacks a fragment of 166 amino acids at the N-terminal region containing a putative leucine zipper domain (30). However, despite this similarity, OPTN is (unlike NEMO) not a regulatory subunit of the IκB kinase (IKK) complex that is essential for NF-κB activation (29). OPTN was shown to block the ability of NEMO to bind ubiquitinated receptor-interacting protein kinase 1 (RIPK1), which resulted in a suppression of TNF-induced NF-κB signaling (8). Similarly, OPTN also inhibits NF-κB signaling through interaction with CYLD that leads to deubiquitination of NEMO and RIPK1 (31). Notably, OPTN is induced by TNF receptor signaling and can thus function as a negative-feedback regulator for NF-κB (32). As such, OPTN negatively regulates TNFα-mediated NF-κB signaling which is critically involved in the regulation of immune responses and cell death signaling. In contrast, *in vivo* experiments using *Optn* knock-out and *Optn^470T^* knock-in mice suggest that OPTN plays no role in the regulation of NF-κB signaling (33, 34).

Furthermore, OPTN was shown to be regulated by and control type I IFN responses (7, 29, 35). Production of type I IFNs is the primary response to bacterial and viral infections (36). Specifically, upon recognition of pathogen-associated molecular patterns by toll-like receptors or RIG-I-like receptors, IFN regulatory factor 3 (IRF3) becomes phosphorylated by TANK-binding kinase 1 (TBK1) and translocates to the nucleus, which leads to the transcription of type I IFN response genes (7). OPTN binds to TBK1 to support IRF3 activation and production of type I IFNs (34). In contrast to this notion, however, OPTN was shown to suppress virus-induced IRF3 signaling (37).

More recently, OPTN was identified as a selective autophagy receptor required for autophagic clearance of *Salmonella enterica* (38), removal of damaged mitochondria (39) and degradation of protein clusters at the ER (24). Selective autophagy receptors, i.e., OPTN, NDP52, p62, and TAX1BP1 recognize ubiquitinated cargo and link it to the autophagosomal membrane (3). Before autophagic clearance of the ubiquitinated cargo, TBK1 activates OPTN by phosphorylation in order to enhance its binding capacity to LC3, a conjugate of the autophagosomal membrane (38, 40). A similar mechanism has for example been demonstrated for mitophagy (41). Only recently, a mechanistic link between OPTN and autophagy has been provided. Bansal and colleagues demonstrated an interaction of OPTN with the core autophagy machinery forming around ATG16L1. More specifically, the authors demonstrated that OPTN was required for the recruitment of the ATG12/ATG5/ATG16L1 complex to phagophores for autophagosomal elongation in starvation-induced autophagy (42).

## The Selective Autophagy Receptor OPTN in Disease

### OPTN Is Required for Pathogen Clearance and an Inflammatory Response in Macrophages

The Segal group and colleagues contributed to our understanding of OPTN in intestinal disease processes (15, 43). Bone marrow-derived macrophages from OPTN-deficient mice exhibited a decreased capacity to respond with TNF-α and IL-6 secretion upon stimulation with heat-inactivated *Escherichia coli*. Defective bacterial handling in OPTN-deficient macrophages was paralleled by a more severe *Citrobacter rodentium*-induced colitis and *E. coli* peritonitis. The more severe phenotype may be explained by an inappropriate immune response at the site of infection which increased mortality of OPTN-deficient animals in both models (43). In line with this, OPTN-deficient HeLa cells exhibited a bacterial handling defect after *Salmonella* infection (38) similar to a more severe phenotype after *Salmonella* infection in mice that was independent from NF-κB or type I IFN responses in macrophages (33). Of note, activation of inflammatory signaling cascades may be determined by distinct ubiquitin chains on bacteria as it was recently shown for the ubiquitin coat on S. *typhimurium* that provides a platform for NF-κB (44). Activation of NF-κB resulted in secretion of pro-inflammatory cytokines and reduced bacterial proliferation (44).

Collectively, these data demonstrate that OPTN limits bacterial infection in the intestine likely by mediating selective autophagy and pathogen clearance.

### Macrophage OPTN Expression Is Reduced in a Proportion of CD Patients

The Segal group also analyzed monocyte-derived macrophages from ~40 patients with CD and UC. They noted that—similar to their findings in OPTN-deficient mice—CD macrophages exhibited impaired immune responses (i.e., TNF-α and IFN-γ secretion) upon stimulation with inactivated *E. coli* when compared to healthy controls which could not be explained by the transcriptional profile (15). The authors identified a CD subgroup which composed 10% of their cohort that expressed reduced *OPTN* in macrophages. Reduced expression may be explained by genetic variation as the authors observed an association with a single nucleotide polymorphism rs12415716 that exhibited a minor allele frequency of ~18%. Indeed, siRNA silencing reduced the inflammatory response of OPTN-deficient macrophages by 25% (43). These two studies highlight that OPTN is required for an appropriate immune response of macrophages upon exposure to bacterial antigens. Whether reduced OPTN expression and an impaired cytokine response is a cause of or consequence from IBDs deserves further attention. Furthermore, it will be interesting to decipher how OPTN modulates immune responses and how this may be related to autophagic processes (43).

### OPTN Limits the Accumulation of an ER-Based Inflammatory Signaling Hub in Intestinal Epithelial Cells

**Figure 1:**
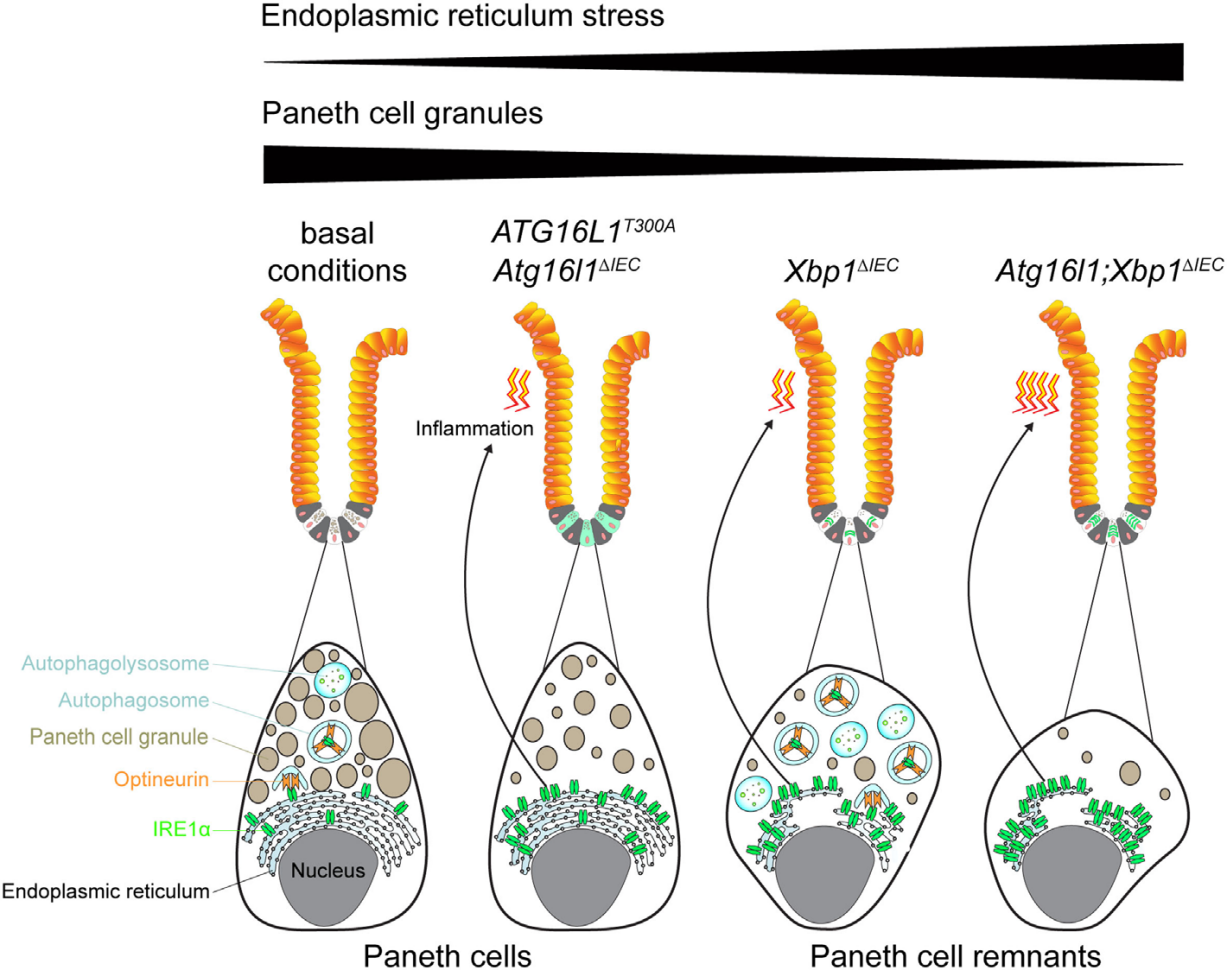
Optineurin (OPTN)-dependent degradation of inositol-requiring enzyme 1α (IRE1α) is abolished in autophagy-deficient Paneth cells resulting in a Crohn’s disease (CD)-like inflammation. Under basal conditions, the endoplasmic reticulum (ER) stress sensor IRE1α is recognized by the selective autophagy receptor OPTN and is subsequently engulfed by the autophagosomal membrane and targeted for degradation in the autophagolysosome. In individuals harboring a homozygous ATG16L1^T300A^ mutation or in mice with an *Atg16l1* deletion in the intestinal epithelium (*Atg16l1^ΔIEC^*), autophagosomes cannot be formed and hence display hypomorphic autophagy. As a result of the defective autophagy as well as a decreased capacity of the unfolded protein response (UPR) with age, ER stress, and IRE1α are accumulating in Paneth cells, which concomitantly leads to the development of a CD-like inflammation in the ileum when mice become older. ATG16L1 is also involved in Paneth cell granule exocytosis and hence integrity is disturbed in ATG16L1-deficient individuals. Mice with a deletion of the UPR transcription factor *Xbp1* in the intestinal epithelium (*Xbp1^ΔIEC^*) also exhibit elevated levels of ER stress and IRE1α, but which are counteracted by increased formation of autophagosomes and OPTN-mediated degradation of IRE1α. In *Atg16l1;Xbp1^ΔIEC^* mice in which both compensatory mechanisms (UPR and autophagy) fail, unrestrained IRE1α leads to the development of a CD-like inflammation similar to the *Atg16l1^ΔIEC^* mice, but earlier in life. Due to prolonged ER stress Paneth cell integrity in *Xbp1^ΔIEC^* and *Atg16l1;Xbp1^ΔIEC^* mice is massively disturbed lacking an expansion of the ER and lacking lysozyme expression due to minuscule Paneth cell granules.

The ER is a cellular organelle which hosts protein synthesis and folding and which instigates trafficking for secretory purposes (45). These processes are fundamentally important for cellular homeostasis which is why they are tightly controlled by redundant mechanisms. One of these mechanisms is the unfolded protein response (UPR) that is equipped with three major sensors of stress at the ER: inositol-requiring enzyme 1, protein kinase RNA-like endoplasmic reticulum kinase, and activating transcription factor 6. These sensors are engaged upon accumulation of unfolded, misfolded, and aggregating proteins in the ER, a condition termed endoplasmic reticulum stress (ER stress) (46). The UPR generally aims at the resolution of ER stress; however, unabated stress at the ER may instigate inflammatory (danger) signaling (47–49). This may be executed for example by the formation of inositol-requiring enzyme 1α (IRE1α) oligomers which cluster and may not be suitable for proteasomal degradation (23, 24). IRE1α is expressed in intestinal epithelial cells and particularly Paneth cells that heavily rely on the UPR due to a high secretory burden (17, 24). IRE1α is a transmembrane receptor that harbors a kinase and endoribonuclease domain, which allows splicing and activation of the transcription factor X-box-binding protein 1 (*Xbp1*) to instigate the UPR and maintain ER homeostasis (50). In turn, unabated ER stress induced by genetic deletion of *Xbp1* in *Xbp1^ΔIEC^* mice hyperactivates IRE1α (23, 51, 52). Importantly, ER stress-induced IRE1α hyperactivation is restrained by autophagy as co-deletion of autophagy-related 16-like 1 (*Atg16l1*) increased the level of IRE1α activation (Figure 1) (23). Defective removal and hyperactivation of IRE1α in *Atg16l1;Xbp1^ΔIEC^* mice was paralleled by a spontaneous CD-like inflammatory phenotype restricted to the small intestine with a fissuring transmural character (23). Indeed, genetic co-deletion of IRE1α ameliorated CD-like inflammation in *Atg16l1;Xbp1^ΔIEC^* mice (24) demonstrating that autophagy-restricted IRE1α activity critically controlled inflammation that emanated from intestinal epithelial cells (23, 24). In these studies, the selective autophagy receptor OPTN emerged as critical regulator of IRE1α degradation in the setting of unabated ER stress (24). These observations suggest that OPTN targets IRE1α, possibly by a ubiquitin signal (53, 54), for autophagosomal degradation to remove an inflammatory signaling hub from the stressed ER (Figure 1). Notably, CD patients harboring the ATG16L1 T300A autophagy-deficient variant (22) exhibited increased IRE1α accumulation in Paneth cells (24), a site of epithelial ER stress (23, 55). Collectively, these data suggest that autophagy controls ER stress and inflammation specifically in Paneth cells of CD patients harboring the ATG16L1^T300A^ variant. This notion is supported by a model of Paneth cell-specific ER stress which led to the development of a spontaneous enteritis (23). In summary, these studies suggest that IRE1α degradation is dependent on ATG16L1-mediated autophagy and possibly on OPTN as an autophagy receptor (24, 42). These data advocate a role for OPTN in inflammatory processes consequent to ER stress, but further studies are needed to corroborate a role for OPTN and selective autophagy during intestinal inflammatory processes.

## Discussion

Optineurin controls autophagic processes by selectively targeting ubiquitinated molecules for autophagic degradation (3, 4). A direct interaction of OPTN with IRE1α in intestinal epithelial cells and the requirement of OPTN for the removal of this inflammatory signaling hub may set a basis for our understanding of how autophagy can resolve ER stress-induced inflammation. We suggest that IRE1α is targeted by OPTN for autophagosomal degradation under conditions of ER stress to restrain IRE1α-mediated danger signaling and inflammation (24). Similarly, ER-phagy of stressed ER membranes also leads to the resolution of ER stress (56) which suggests once more that a tight control of the ER is indispensable for cellular homeostasis (57–61). Furthermore, OPTN is required to target a critical autophagy hub containing ATG16L1 to the forming phagophore (42). However, we acknowledge that distinct mechanisms other than selective autophagy may control IRE1α activity and an inflammatory threshold (62).

Understanding the selectivity of OPTN-mediated autophagy would be highly informative. For example, definition of an unbiased OPTN interactome could help to understand two major biological functions of OPTN (i.e., selective autophagy and regulation of inflammatory pathways) and to define their relationship in health and disease. More specifically, it may be critical to discriminate OPTN-mediated autophagy functions from those that are independent of autophagy as it currently unclear how they are interconnected. Some literature would support the notion that receptors of selective autophagy are critically involved in inflammatory processes (63) similar to a genetic variant in *NDP52* with CD (64). Furthermore, autophagy receptors may control microbial dissemination (65, 66), a concept that becomes increasingly relevant in dysbiotic situations as seen in IBD (67). Reduction of *OPTN* expression in macrophages of some CD patients may not just result in diminished cytokine secretion upon bacterial infections (15), but may also lead to a decreased autophagic containment of pathogens (or commensals) and degradation of inflammatory molecules as exemplified for IRE1α in Paneth cells (24). However, it may well be that microbial control and ER stress are interrelated pathways (68). The impact of these observations in IBD deserve further attention and direct evidence for the regulation and function of OPTN in epithelial cells of CD patients is eagerly awaited.

In case of OPTN-mediated autophagy, we are only beginning to appreciate a role in intestinal inflammatory disease processes. However, we propose broad implications for OPTN in ER stress-related diseases within and beyond the intestine (24, 69–71). A driving force (besides genetic variation and environmental cues) may be cellular senescence with a declining capacity of the UPR during aging (72).

## Concluding Remarks

Evidence accumulates for a role of OPTN in disease processes within and beyond the intestine. Direct evidence for a role of OPTN in CD is limited. However a concept arises, in which OPTN is required for the removal of inflammatory molecules from the ER and invading bacteria (14, 15, 24), which may be governed by OPTN-mediated selective autophagy. As such, OPTN emerges as a critical link between ER disturbances and the resolution by autophagy (24). This observation is of note as ER stress is commonly observed in IBD patients and especially those harboring prominent genetic risk factors (e.g., ATG16L1 and NOD2) (22, 73, 74), which reflects one facet in this complex inflammatory condition (17, 19). Pharmacologic targeting of autophagy may indeed be beneficial in IBD which could depend on the ability of the host to launch an appropriate autophagic response (75). Moreover, clinically established drugs may exert their beneficial effects through modulation of autophagy (75). As such, understanding the biology of OPTN in CD may help to establish or guide future therapies.

## Author Contributions

MT and TA contributed equally to this manuscript.

## Conflict of Interest Statement

The authors declare that the research was conducted in the absence of any commercial or financial relationships that could be construed as a potential conflict of interest.
